# Single-Center Investigation of Brain Injury Guidelines (BIG)-3 Classification in Traumatic Brain Injury: A Retrospective Analysis of the Role of Pre-injury Antithrombotic Therapy

**DOI:** 10.7759/cureus.109820

**Published:** 2026-05-28

**Authors:** Munir Shunnar, Zubair Ahammad, Amer A Afaneh

**Affiliations:** 1 Emergency Medicine, Mercy Health, Toledo, USA; 2 Neurosurgery, St. Vincent's Medical Center, Toledo, USA; 3 General Surgery, Bon Secours Mercy Health St. Vincent Hospital, Toledo, USA

**Keywords:** blood thinner, brain bleed, head and neck trauma, surgical-trauma icu, traumatic brain injury

## Abstract

Background

Traumatic brain injuries (TBIs) are sometimes classified using the Brain Injury Guidelines (BIG) into categories BIG-1, BIG-2, and BIG-3, which determine management strategies. The BIG is a validated tool that triages patients with TBI into those who can be safely managed by the trauma service and those who require neurosurgical consultation and repeat imaging. Patients receiving pre-injury antithrombotic therapy, including anticoagulants and antiplatelet agents, are commonly upgraded to higher BIG classifications despite otherwise low-risk clinical and imaging features. This study evaluates whether pre-injury antithrombotics alone warrant BIG-3 classification and assesses associated clinical outcomes.

Methods

We conducted a retrospective analysis of TBI patients classified as BIG-3 solely due to antithrombotic use, with no other BIG-3 criteria met. We selected patients who would have otherwise been a BIG-1 had it not been for antithrombotics. Data were collected from 8/19/2019 to 7/24/2024, including demographics, injury characteristics, antithrombotic regimen, and clinical outcomes, at Bon Secours Mercy Health St. Vincent Hospital, Toledo, USA. Pre-injury antithrombotic therapy was defined as documented use of anticoagulant agents (e.g., warfarin, apixaban, and rivaroxaban) or clinically significant antiplatelet therapy, including dual antiplatelet regimens (e.g., aspirin and clopidogrel), at the time of injury. Patients receiving low-dose aspirin monotherapy (“baby aspirin”) were excluded from the study cohort. Primary outcomes included CT progression, neurological decline, neurosurgical intervention, and mortality. Data are presented as numbers and percentages or means ± standard deviations.

Results

A total of 375 patients were screened, of whom 69 patients (18.4%) met the inclusion criteria, with a mean age of 77.9 years and 50.7% female. Falls were the predominant mechanism of injury, accounting for 64 cases (92.8%). Sixty-one patients (88.4%) presented with a Glasgow Coma Scale (GCS) score of 15. Thirty-three patients (47.8%) were receiving single-agent therapy, and 36 patients (52.2%) were receiving dual therapy, with aspirin and clopidogrel being the most common combination (43.5%). None required intubation, had skull fractures, or were intoxicated. CT progression occurred in only two patients (2.9%), both of whom remained neurologically stable and did not require neurosurgical intervention. Three patients (4.3%) required intensive care admission. There were no cases of neurological decline or surgical intervention. One patient (1.5%) died, but the death was unrelated to brain injury.

Conclusion

In our single-center retrospective cohort of 69 patients, we found that anticoagulated TBI patients, who would have otherwise been classified as BIG-1, had minimal CT progression, no neurological decline, and no neurosurgical interventions. These findings support further investigation and a potential area of study for prospective validation.

## Introduction

Traumatic brain injury (TBI) remains a major global public health concern, with an estimated 69 million new cases occurring annually worldwide [1]. In the United States alone, TBI accounts for more than 2.8 million emergency department visits, hospitalizations, or deaths each year, contributing to substantial healthcare expenditures exceeding $76 billion when direct medical costs and lost productivity are considered [2]. The increasing availability and sensitivity of computed tomography (CT) imaging have led to greater detection of small intracranial hemorrhages, particularly among older adults presenting after low-energy mechanisms, such as ground-level falls. These clinical scenarios frequently raise an important management dilemma regarding which patients require intensive monitoring, repeat imaging, and neurosurgical consultation, and which can be safely managed with conservative observation.

To address this challenge, the Brain Injury Guidelines (BIG) were originally developed by Joseph et al. as a structured triage framework for patients with mild TBI and intracranial findings on CT imaging [3]. The BIG algorithm categorizes patients into BIG-1, BIG-2, or BIG-3 groups based on neurological examination findings, radiographic hemorrhage characteristics, and specific risk modifiers, such as intoxication, skull fracture, and preinjury antithrombotic use. In a subsequent prospective multi-institutional validation study involving 2,432 patients across nine Level I and Level II trauma centers, the implementation of BIG was associated with a reduction of approximately 29% in repeat head CT imaging and neurosurgical consultations without missed operative lesions [4]. These findings established BIG as a valuable triage tool that is cost and resource-effective while maintaining patient safety.

Despite its demonstrated utility, a central component of the BIG algorithm remains controversial. The current guidance mandates automatic classification of patients receiving preinjury anticoagulation or dual antiplatelet therapy into the highest risk category, BIG-3, regardless of otherwise favorable clinical and radiographic features [3,4]. This conservative approach reflects longstanding concerns regarding delayed hemorrhagic expansion in anticoagulated patients and the potentially catastrophic consequences of missed progression. However, emerging evidence suggests that the risk associated with antithrombotic exposure is heterogeneous and influenced by factors, including anticoagulant class, hemorrhage size and morphology, patient age, and the reliability of neurological examination [5-8]. As direct oral anticoagulants have increasingly replaced warfarin and dual antiplatelet therapy has become more common in the aging population, the clinical relevance of blanket escalation based solely on medication exposure warrants further study.

At our institution, a modified BIG pathway is utilized that incorporates Glasgow Coma Scale (GCS) thresholds and more specific hemorrhage size criteria [9]. Modified BIG frameworks have been evaluated in multicenter studies demonstrating safe reductions in resource utilization while maintaining clinical outcomes [5]. No prior study at our center has specifically evaluated outcomes among patients upgraded to BIG-3 classification solely due to antithrombotic exposure despite otherwise meeting BIG-1 criteria, including normal neurological examination, absence of intoxication or skull fracture, and minimal intracranial hemorrhage on initial CT imaging [10]. Given the increasing prevalence of anticoagulant and antiplatelet use among elderly trauma patients, clarifying the true risk profile of this subgroup is of growing clinical importance.

The objective of the present study was to perform a retrospective analysis of adult blunt TBI patients classified as BIG-3 exclusively due to anticoagulation exposure while otherwise meeting the BIG-1 criteria. We evaluated clinical outcomes, including radiographic progression on repeat CT imaging, neurological deterioration, neurosurgical intervention, intensive care utilization, and mortality. We hypothesized that anticoagulation exposure alone may not confer the risk of deterioration in otherwise low-risk patients with mild TBI. The findings of this study aim to inform ongoing refinement of BIG and modified BIG algorithms and contribute to evidence-based optimization of resource utilization while maintaining patient safety.

## Materials and methods

Study design and setting

This retrospective cohort study was conducted at Bon Secours Mercy Health St. Vincent Hospital, a Level I trauma center in Toledo, USA, under Institutional Review Board exemption (Routine Progression Protocol RP3, Exempt Category 4, with a Health Insurance Portability and Accountability Act (HIPAA) waiver). Eligible patients were identified through retrospective review of the institutional trauma registry and electronic medical records using diagnosis codes consistent with traumatic intracranial hemorrhage (ICD-10 codes S06.xx), combined with medication reconciliation records indicating pre-injury antithrombotic therapy.

Statistical methods

Data are described with frequency counts and percentages. Age is shown with mean and standard deviation. Data were analyzed with SAS version 9.4 (SAS Institute Inc., Cary, NC).

Study population

Adult patients aged 18 years or older presenting with blunt TBI between August 2019 and July 2024 were screened using the institutional trauma registry and electronic medical record system.

Inclusion criteria

Patients were included if they were classified as BIG-3 solely due to preinjury anticoagulation or dual antiplatelet therapy. They had to fulfill the other modified BIG-1 classification requirements, including a GCS score of 14 to 15 on presentation, a demonstrated normal neurological examination, no evidence of intoxication or skull fracture, and a single small intracranial hemorrhage on initial CT imaging consistent with BIG-1 classification. BIG-1 Bleed Characteristics area as follows: subdural hematoma (SDH) ≤ 4 mm, no epidural hematoma (EDH), intraparenchymal hemorrhage (IPH) ≤ 4mm, or trace subarachnoid hemorrhage (SAH).

Exclusion criteria

Patients were excluded if they had focal neurological deficits, met BIG-2 or BIG-3 criteria unrelated to anticoagulation exposure, were intoxicated at presentation, sustained penetrating trauma, had incomplete imaging or missing clinical data, were younger than 18 years, or were receiving only low-dose aspirin monotherapy.

Imaging protocol

All patients underwent an initial non-contrast head CT scan followed by repeat imaging within six hours according to institutional BIG-3 protocol.

Outcomes

Primary outcomes included radiographic progression on repeat imaging, neurological deterioration during hospitalization, need for neurosurgical intervention, intensive care unit (ICU) admission, and in-hospital mortality.

Statistical analysis

Given the descriptive nature of the study and the absence of comparative hypothesis testing, no inferential statistical tests were performed.

Descriptive statistics were generated using SAS version 9.4. Continuous variables are reported as mean plus or minus standard deviation, and categorical variables are reported as number and percentage.

## Results

**Figure 1:**
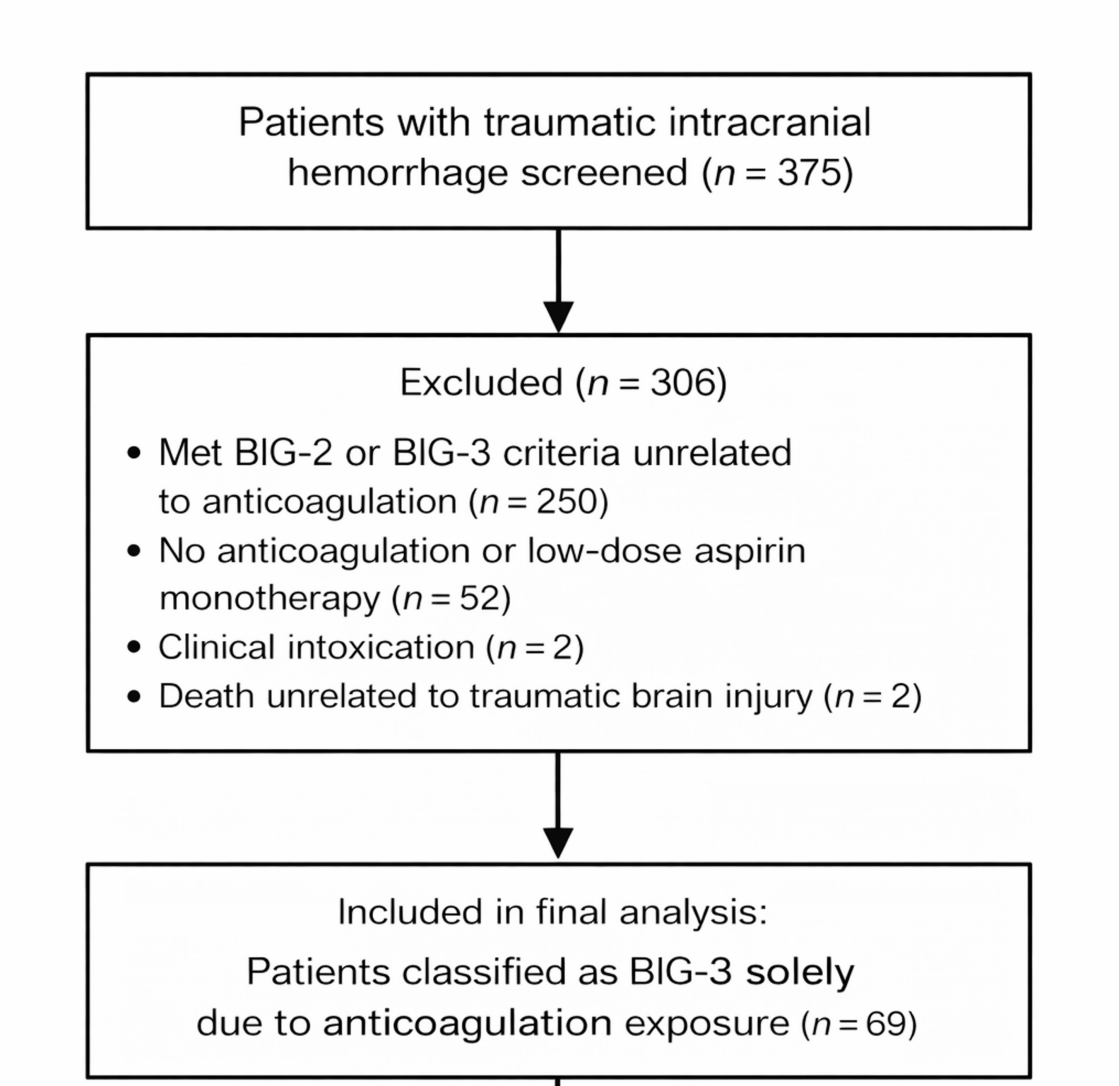
Cohort flow diagram demonstrating patient selection Of 375 screened patients with traumatic intracranial hemorrhage, 306 were excluded due to alternative BIG-2 or BIG-3 criteria, absence of anticoagulation exposure, intoxication, or death unrelated to traumatic brain injury. A final cohort of 69 patients met the inclusion criteria. This image was created using Microsoft PowerPoint (Microsoft® Corp., Redmond, WA).

A total of 375 patients were screened. After applying exclusion criteria, 69 patients (18.4%) were included in the final cohort. The predominant reason for exclusion was hemorrhage burden or imaging features inconsistent with BIG-1 classification, most frequently reflecting larger intracranial hemorrhage size or intoxication. Additional exclusions were related to abnormal neurological examination findings. Of these, 306 were excluded, including 250 patients who met the BIG-2 or BIG-3 criteria unrelated to anticoagulation, 52 patients without anticoagulation or receiving low-dose aspirin monotherapy, two patients with clinical intoxication, and two patients who died of non-traumatic causes during admission. The remaining 69 patients (18.4%) met the inclusion criteria (Figure 1).

The mean age was 77.9 ± 11.7 years, and 35 patients (50.7%) were female. Falls represented the predominant mechanism of injury in 64 patients (92.8%). Sixty-one patients (88.4%) had a GCS score of 15. 

**Figure 2:**
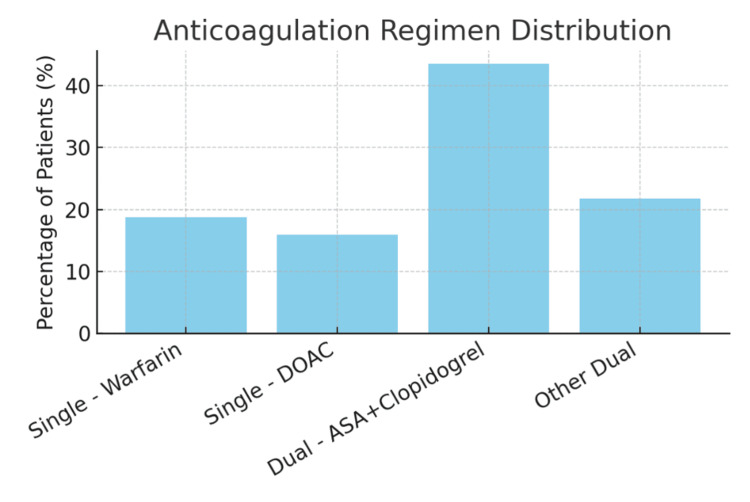
Anticoagulation regimen distribution Bar graph displaying the proportion of patients on single vs. dual anticoagulation therapy, with further breakdown by specific agent type. Thirty-three patients (47.8%) were receiving single-agent therapy, while 36 patients (52.2%) were receiving dual therapy. Data are presented as numbers and percentages (n, %). No statistical comparisons were conducted. ASA, acetylsalicylic acid; DOAC, direct oral anticoagulant The image was created using Microsoft PowerPoint (Microsoft® Corp., Redmond, WA).

Initial CT scans revealed SDH in 42 patients (60.9%), trace SAH in 24 patients (34.8%), and small IPH in three patients (4.4%) (Table 1). All patients underwent repeat CT within six hours. Radiographic progression was identified in only two patients (2.9%), both of whom remained neurologically intact and required no neurosurgical intervention (Figure 2).

**Table 1:** Radiographic findings Subdural hematoma was identified in 42 patients (60.9%), trace subarachnoid hemorrhage in 24 patients (34.8%), and small intraparenchymal hemorrhage in three patients (4.4%). Data are presented as numbers and percentages (n, %). No statistical comparisons were conducted.

Finding	n (%)
Subdural hematoma (SDH)	42 (60.9)
Trace subarachnoid hemorrhage (SAH)	24 (34.8)
Intraparenchymal hemorrhage (IPH)	3 (4.4)

ICU admission occurred in three patients (4.3%), all for precautionary observation rather than deterioration. The mean hospital length of stay was 2.1 ± 1.3 days. There were no cases of neurological decline during admission. One patient (1.5%) died during hospitalization due to causes unrelated to TBI. Radiographic progression was rare and occurred in one patient receiving a direct oral anticoagulant (DOAC) and one patient receiving acetylsalicylic acid (ASA) plus clopidogrel, while no progression was observed among patients receiving warfarin or other anticoagulation therapies. No formal statistical comparisons were performed.

## Discussion

In our retrospective, single-center cohort of 69 patients, we found a low rate of radiographic progression and an absence of clinically significant neurological deterioration among patients classified as BIG-3 solely due to preinjury anticoagulation exposure despite otherwise favorable clinical and radiographic features. These findings contribute to a growing body of literature suggesting that anticoagulation status alone may not warrant high-risk classification in patients with mild TBI [4-8]. This study is not sufficient to change our practice of modified BIG; however, its findings were interesting and raise opportunities for further research. 

The BIG was developed as a pragmatic triage framework to streamline management decisions and reduce unnecessary neurosurgical consultation and repeat imaging [3]. Subsequent prospective multi-institutional validation demonstrated that implementation of the guidelines was associated with meaningful reductions in resource utilization without missed operative lesions [4]. However, the automatic escalation of all anticoagulated patients to the highest risk category reflects a historically conservative approach rooted in concerns regarding delayed hemorrhagic progression and catastrophic neurological decline [3,4].

Emerging evidence suggests that the risk associated with antithrombotic exposure is heterogeneous and influenced by anticoagulant class, hemorrhage size and morphology, patient age, and the reliability of neurological examination [7-9,11]. Observational studies have reported variable rates of intracranial hemorrhage progression among anticoagulated patients, with some data suggesting that direct oral anticoagulants may not confer uniformly higher risk compared with warfarin [9,12]. In addition, antiplatelet therapy, particularly dual antiplatelet regimens, has been associated with increased risk in certain clinical contexts, although findings remain inconsistent across studies [7,8]. 

An important consideration highlighted by this study is the distinction between anticoagulant and antiplatelet therapy in the risk stratification of traumatic intracranial hemorrhage. While current escalation practices often group these therapies under a single high-risk category, their pharmacologic mechanisms and associated bleeding risks differ. Our findings suggest that stable patients on pre-injury antithrombotic therapy, particularly those with preserved neurological examination and low-risk imaging features, may not uniformly require escalation to higher levels of monitoring. 

In the present cohort, radiographic progression occurred in only a small proportion of patients and did not result in neurosurgical intervention or neurological decline. These findings suggest a potential area of further investigation with regard to the need for routine re-imaging. Prior investigations evaluating the value of routine repeat CT imaging in neurologically stable patients with mild TBI have similarly questioned its impact on clinical management [13,14]. Studies have demonstrated that a single antiplatelet regimen does not increase the risk of worse outcomes, whereas dual antiplatelet therapy is associated with an increased risk of bleeding [15]. Our study did not include patients receiving only a single antiplatelet agent (e.g., ASA 325 mg or clopidogrel); however, this would be another area worth investigating.

Given the increasing prevalence of anticoagulant and antiplatelet use among elderly trauma patients, refinement of risk stratification protocols represents an important opportunity to improve care efficiency while maintaining patient safety [6-8]. Future prospective multicenter investigations incorporating anticoagulant class, laboratory markers, platelet function assessment, and detailed radiographic characteristics will be essential to inform evidence-based updates to existing management guidelines.

Limitations 

This study has several limitations. Its retrospective design introduces the potential for selection bias and unmeasured confounding. Additionally, this was a single-center analysis conducted at a Level I trauma center with established trauma service protocols, which may limit generalizability to institutions with different practice patterns or patient populations. The relatively small sample size of patients meeting the inclusion criteria may also reduce the power to detect rare adverse outcomes. This small sample size limits the ability to make any changes to the existing guidelines. Furthermore, management decisions were not protocolized and may have been influenced by individual provider discretion. Fourth, the distribution of antithrombotic regimens was weighted toward antiplatelet therapy, which may not fully reflect risk profiles associated with other anticoagulant classes. In addition, we did not look at the international normalized ratio (INR) or thromboelastogram (TEG) of these patients. This may have been clinically relevant, for example, in a patient on warfarin who presents sub-therapeutic. Finally, outcomes were limited to in-hospital follow-up, and delayed complications after discharge were not assessed. Prospective multicenter studies are needed to validate these findings and better define the role of pre-injury antithrombotic therapy in BIG classification.

## Conclusions

Among carefully selected patients with mild TBI and otherwise low-risk clinical and radiographic features, anticoagulation exposure alone did not lead to clinically significant bleed progression. Individualized risk stratification strategies that consider medication class, injury characteristics, and clinical presentation may improve resource utilization without compromising patient safety. Further prospective and larger studies are warranted to investigate the implications of anticoagulation use in patients with otherwise mild TBI.
